# DNA Methylation, SERPING1 Expression, and Immune-related Traits in Osteoporosis: A Mendelian Randomization Study And Supportive *Ex Vivo* Evidence

**DOI:** 10.2174/0118715303462423260125161230

**Published:** 2026-03-09

**Authors:** Shangtong Chen, Tian Xia, Chuanhong Huang

**Affiliations:** 1 School of Orthopedics, Guangxi University of Chinese Medicine, Nanning, Guangxi, 530000, China;; 2 Department of Orthopedics, Ruikang Hospital affiliated to Guangxi University of Chinese Medicine, Nanning, Guangxi, 530000, China

**Keywords:** DNA methylation, SERPING1, osteoporosis, immune cells, epigenetic regulation, bone disorder

## Abstract

**Introduction:**

Osteoporosis (OP) is a major public health burden; however, the role of SERPIN family proteins remains incompletely understood. This study aimed to investigate genetically inferred associations between SERPINs and OP and to explore potential regulatory relationships.

**Methods:**

Genome-wide association study (GWAS) summary statistics were used to perform two-sample Mendelian randomization (MR) and summary-data-based Mendelian randomization (SMR) analyses. Primary MR estimates were derived using inverse-variance-weighted (IVW), MR-Egger, weighted median, simple mode, and weighted mode methods. Cancellous bone tissue from the greater trochanter of the femur was collected from three patients with OP and three non-osteoporotic controls. qPCR and WB were used to analyze the whole bone homogenate, IHC was used to detect decalcified bone sections, and mediation analysis was used to explore potential regulatory associations.

**Results:**

Among the proteins of the SERPIN family, only SERPING1 had an obvious positive correlation with the risk of osteoporosis (OR = 1.06, *P* = 0.0012). qPCR, WB, and IHC analyses demonstrated increased SERPING1 mRNA and protein expression in bone tissue from OP patients. Mediation analyses suggested that cg15918732 DNA methylation may serve as an upstream regulatory factor for SERPING1 expression. In addition, the downstream associations also consist of the alteration of immune cell conditions, like the decrease in the quantity of natural killer cells and T cells, and the rise in the level of mononuclear cells, and so forth.

**Discussion:**

These findings provide genetic evidence for the possible role of SERPING1 in OP, which may be achieved through epigenetic regulation and immune pathways. Due to the corresponding characteristics of genetic inference and the small sample size, these results are hypothetical and generative.

**Conclusion:**

This study shows that DNA methylation of cg15918732 may be associated with SERPING1 expression in osteoporosis and immune-related traits. These findings provide new insights into potential epigenetic and immunological pathways in osteoporosis, which may contribute to future mechanistic and translational research.

## 1 INTRODUCTION

Osteoporosis (OP) is a systemic metabolic bone disorder characterized by reduced bone mineral density, deterioration of bone microarchitecture, and increased bone fragility, all of which collectively heighten the risk of pathological fractures [1-3]. An epidemiological study has reported that the prevalence of OP in China mainland is 5.0% among men and 20.6% among women aged 40 years and older [4]. The prevalence of osteoporosis (OP) in men aged 50 and above in South Korea is 8.8% [5]. The latest report from the International Osteoporosis Foundation points out that about 1/3 of women and 1/5 of men over 50 years old will have osteoporotic fractures in their lifetime [6]. Osteoporotic fractures lead to a large number of morbidities and are also the main factors causing disability and death in the elderly [7]. Within 1 year after hip fracture, about 20% of patients die of related complications, and nearly 50% of patients are in long-term disability and have reduced quality of life [8]. A small number of people with disabilities recover to the activity level before fracture, and most people need long-term care, which shortens the healthy life expectancy [9]. A large amount of evidence shows that the physiological bone balance needs to be adjusted to osteoblast bone formation and osteoclast bone resorption [10, 11], among which osteoprotegerin derived from B cells can inhibit osteoclast generation [12, 13]. However, in pathological conditions, this balance is broken—especially when bone resorption exceeds bone formation, then osteoporosis will come into being [14]. When both inflammation and bone metabolism disorder coexist, activated T cells and B cells will secrete a series of osteoclastogenesis factors, like receptor activator of nuclear factor-κB ligand (RANKL), interleukin-17A (IL-17A), and tumor necrosis factor-α (TNF-α). These mediators, which can cause excessive activation of osteoclasts, will then result in bone resorption being more than bone formation. As a result, this kind of imbalance will trigger or aggravate osteoporosis and bring about such a vicious cycle where bone loss and chronic inflammation enhance each other [15]. Even so, the specific pathogenic mechanisms underlying OP have not yet been fully elucidated.

Serine protease inhibitors (SERPINs) are a large superfamily. Carrell proposed in 1985 that they have a conserved primary structure [16]. To date, 36 SERPIN proteins have been identified in humans. Their typical structure comprises an N-terminal helical domain of approximately 400 amino acid residues, a C-terminal β-barrel domain, and a critical reactive center loop (RCL). The RCL can insert into β-sheet A to form an extremely stable conformation that underlies the inhibitory mechanism of SERPINs. Members of the SERPIN family are related to a variety of diseases. Antithrombin (AT) and plasminogen activator inhibitor-1 (PAI-1) are crucial in the process of hemostasis and coagulation, and are also related to thrombotic diseases such as stroke and venous thrombosis [17, 18]. C1 esterase inhibitor (C1-INH), encoded by the SERPING1 gene, regulates the classical complement pathway and the mannose-binding lectin (MBL) pathway by inhibiting the proteases C1r/C1s and MASP-1/MASP-2, respectively. Clinically, C1-INH is widely used in the treatment of hereditary angioedema [19]. Members of the SERPIN family are also involved in many orthopedic diseases. For example, a bioinformatics study found that SERPINH1 was significantly up-regulated in samples of hormone-induced osteonecrosis of the femoral head [20]. PAI-1 has been implicated in the reduction of bone mineral density (BMD) observed in patients with chronic kidney disease [21] and type 2 diabetes [22]. SERPIN family proteins, as critical upstream regulators, are extensively involved in multiple pathophysiological processes closely linked to OP pathogenesis, including inflammation [23], coagulation [24], angiogenesis [25], and immune cell regulation [26]. However, whether SERPINs have a causal role in OP has not been fully explored. Due to the different functions among members, it is not clear which specific SERPINs play the main role. It is necessary to clarify the relationship between the SERPIN family and osteoporosis to carry out disease research and find new treatment strategies.

DNA methylation belongs to one of the epigenetic mechanisms. It does not change the DNA sequence but can regulate gene expression [27, 28]. This process involves the covalent addition of a methyl group to the 5′ position of cytosine (C), producing 5-methylcytosine (5mC) [29]. This modification mainly occurs at CpG sites and exists in gene promoter regions, gene bodies, and repetitive sequences. It is also involved in key biological processes, such as transcriptional repression, stem cell differentiation, and inflammation regulation [30]. Recent studies have shown that changes in the DNA methylation pattern of specific genomic regions are closely related to the occurrence and development of osteoporosis. In particular, hypomethylation of Alu repetitive elements has been positively correlated with advancing age and is significantly associated with reduced BMD [31]. Hypermethylation of the promoter region of the Bone Morphogenetic Protein 2 (BMP2) gene suppresses its transcriptional activity, thereby impairing osteogenic differentiation [32]. Hypomethylation of the promoter region of the TBC1D8 gene has been associated with an increased risk of OP [33]. In addition, the expression of multiple SERPIN family genes is strictly regulated by DNA methylation. For example, promoter or regulatory region hypomethylation of SERPINE1, SERPINA3, and SERPINA1 has been reported to upregulate gene expression across multiple diseases, including PCOS [34], placental disorders [35], and thyroid cancer [36]. Although previous studies suggest that DNA methylation contributes to OP pathogenesis by regulating the expression of key genes [32, 33], whether it promotes disease development through the SERPIN family remains incompletely understood. Based on the immune regulatory function of SERPIN family members and the role of DNA methylation in bone metabolism, we hypothesize that the epigenetic regulation of specific SERPIN may be related to the susceptibility of OP.

Mendelian randomization (MR) is an emerging analytical approach that employs single-nucleotide polymorphisms (SNPs) as naturally occurring instrumental variables to infer potential causal relationships between exposures and outcomes [37, 38]. This method has been widely applied to identify and evaluate potential risk factors for OP [39-42]. In this study, MR analysis was carried out using cis-protein quantitative trait loci (cis-pQTLs) of the SERPIN family and pQTL data of deCODE to explore the causal relationship between SERPIN proteins and OP. To ensure the reliability of the results, Summary data-based Mendelian randomization (SMR) analysis, quantitative real-time PCR (qPCR), Western blotting (WB), and immunohistochemistry (IHC) analyses were used for verification. Besides, mediation analysis was conducted to investigate the upstream and downstream regulatory mechanisms of the identified SERPIN proteins.

## 2 MATERIALS AND METHODS

### 2.1 Exposure-Related pQTL Dataset

Cis-pQTL refers to genetic variations that have an impact on gene expression or protein abundance within or near the target gene [43]. In this study, the cis-pQTL data of the SERPIN family genes by Zheng *et al*. were used. This data has 738 cis-acting single-nucleotide polymorphisms related to 734 proteins, which can evaluate the variations affecting the levels of circulating proteins [44]. The deCODE database has plasma proteomic and genetic variation data for 4,674 disease-related proteins. It provides a comprehensive resource for studying the genetic determinants of protein expression and their associations with complex diseases [45]. After preliminary screening, the pQTL data of deCODE were used to analyze and revalidate the positive results.

When using Single nucleotide polymorphisms (SNPs) as instrumental variables, the following situations need to be met: (1) SNPs reached genome-wide significance (*P* < 5×10^-5^); (2) SNPs located within the human major histocompatibility complex (MHC) region were excluded to avoid potential confounding; (3) only SNPs located within ±1 Mb of the corresponding gene were retained as cis-acting variants; (4) SNPs in linkage disequilibrium (r^2^ > 0.1) were pruned to ensure independence.

### 2.2 Outcome-Related Dataset

The FinnGen project is a large-scale genomics initiative in Finland that integrates genotype data with nationwide health registry information from approximately 500,000 participants enrolled in the Finnish biobank [46]. The FinnGen R9 dataset represents the ninth release of this project, providing updated and quality-controlled genome-wide association summary statistics across numerous diseases and traits. In this study, the OP outcome dataset was obtained from the FinnGen R9 release (https://storage.googleapis.com/finngen-public-data-r9/summary_stats/finngen_R9_M13_OSTEOPOROSIS.gz). There are 7,300 OP cases and 358,014 healthy controls in the dataset. All participants were of European ancestry. Summary statistics were adjusted for key covariates (age, sex, principal components) to control for population stratification.

### 2.3 Two-sample Mendelian Randomization Analysis

We conducted a two-sample MR analysis by using the TwoSampleMR package (version 0.6.19) in R Studio. We took the proteins with drug-operable potential in the SERPINs family (Zheng *et al*.) as exposure variables and OP as the outcome variable [44]. The estimation method was selected based on the number of instrumental variables. For a single instrumental variable SNP, we used the Wald ratio method; the inverse variance weighting method (IVW) was used. Then, SERPIN family members that exhibited statistically significant causal associations with OP were selected for further analysis. For these proteins, we downloaded the pQTL data from the deCODE database as exposure variables in the second two-sample MR analysis, and took OP as the outcome. We used several MR approaches, including IVW, MR-Egger, weighted median, and both simple and weighted mode analyses to evaluate causal effects. IVW was used as the primary analysis, and other methods were applied as sensitivity analyses to assess potential pleiotropy. If all these methods had consistency in effect direction across, the result was considered robust causal associations [47]. The stability of the results was further evaluated by conducting heterogeneity analyses, pleiotropy assessments, and leave-one-out analyses.

### 2.4 SMR Analysis

SMR analysis is a statistical method that combines GWAS summary statistics and expression quantitative trait locus (eQTL) analysis to evaluate the pleiotropic association between gene or protein expression and complex traits [48]. The heterogeneity in dependent instruments (HEIDI) test in the SMR analysis was used to evaluate potential horizontal pleiotropy. The null hypothesis of the HEIDI test is that there is no horizontal pleiotropy between the exposure factor and the outcome [49]. We used the SMR and HEIDI methods determine whether the effects of SNPs on the phenotype occur through alterations in protein expression or *via* alternative biological pathways. The software tool “smr-1.3.1-win.exe” was downloaded from the eQTLGen Consortium website [50].

### 2.5 Collection of Human Bone Samples

Human bone samples were collected from patients diagnosed with hip fractures who underwent hip replacement surgery. In consideration of the prior use of large-scale datasets in the Mendelian randomization analysis—and following the principles of minimizing patient burden and adhering to the biological rule of triplicate validation—a total of six cases were included in this study. Sample collection was conducted from April to July 2023, and all patients included in the study were male, aged 40-80 years. During surgery, a small amount of cancellous bone was harvested from the apex of the affected femoral trochanter. Among patients with hip fractures, group OP refers to those clinically diagnosed with osteoporosis, and group N-OP refers to those without osteoporosis.

### 2.6 Quantitative Real-Time PCR Analysis

Detection of SERPING1 mRNA levels in human bone by qPCR. Total RNA was extracted from bone using RNA extraction solution (Servicebio, G3013). The concentration and purity of RNA were determined using a NanoDrop 2000 spectrophotometer, and all samples were diluted to 200 ng/μL for subsequent experiments. A 20 μL system was used for the reverse transcription reaction, which contained SweScript All-in-One RT SuperMix for qPCR Kit (Servicebio, G3337). The system contained 4 μL of 5×SuperMix, 1 μL of gDNA Remover, 10 μL of total RNA, and was supplemented to the final volume with nuclease-free water. Followed the manufacturer's instructions for cDNA synthesis. Used 2×Universal Blue SYBR Green qPCR Master Mix (Servicebio, G3326) for Q-PCR operation. Analyzed each cDNA sample with three technical replicates. Each cDNA sample was analyzed in triplicate. The total volume of the reaction system was 15 μL, which contained 7.5 μL of premix, 1.5 μL of primer mix (H - SERPING1(1)-S: ATGCTTTGGTAGATTTCTCCCTG), 2.0 μL of cDNA template, and 4.0 zero microliters of nuclease-free water. After loading the completed sealed reaction plate, a brief centrifugation was performed. Amplification was carried out on the CFX Connect real-time fluorescent quantitative PCR instrument with the conditions: pre-denaturation at 95°C for thirty seconds; denaturation at 95°C for fifteen seconds and annealing/extension at 60°C for thirty seconds, for a total of forty cycles; the melting curve rose from 65°C to 95°C, and fluorescent signals were collected at intervals of 0.5°C to check specificity. The relative expression level of SERPING1 was calculated by 2 ^−^ ΔΔCT. The data were the mean plus or minus the standard deviation. A two-sample t-test was used to determine statistical significance, and a *P* value less than 0.05 was considered statistically significant.

### 2.7 Western Blot

We took about 0.1 grams of bone tissue, washed it 2 to 3 times with pre-cooled PBS, and added lysis buffer (Servicebio, G2002) at ten times the volume of the tissue for homogenization. We centrifuged the homogenate at 12000 rpm for 10 minutes, collected the supernatant, and obtained total protein. We determined the protein concentration using a BCA protein assay kit (Servicebio, G2026). The protein sample was mixed with the loading buffer (Servicebio, G2075). After denaturation by boiling water, separation was carried out by SDS-PAGE (Servicebio, G2075). Then, the protein was transferred to a PVDF membrane (Servicebio, WGPVDF45) pre-activated with methanol. The membrane was blocked with a protein - free rapid blocking buffer (Servicebio, G2052) at room temperature. Incubated with the primary antibodies SERPING1 (Servicebio, GB112165-100, 1:5000) and ACTIN (Servicebio, GB15001, 1:5000) at 4°C overnight. After washing with TBST for 3 times, the membrane was incubated with the corresponding HRP-labeled secondary antibody (Servicebio, GB23301; GB23303) at room temperature for 30 minutes. The protein bands were developed using ECL luminescent solution (Servicebio, G2014; G2020), and the gray values of the bands were quantified using ImageJ software. ACTIN was used as an internal reference control to calculate the relative protein expression level. All quantitative data were expressed as mean ± SD. Statistical analyses were performed using a two-sample t-test, and a difference was considered statistically significant when * P*< 0.05.

### 2.8 Immunohistochemistry

After fixing and decalcifying the bone samples, paraffin embedding and sectioning work were carried out. The paraffin sections were placed in environmental protection dewaxing solutions I, II, III (Servicebio, G1128) and incubated for 10 minutes, respectively, to perform gradient dewaxing, then dehydration operations were carried out for 5 minutes, respectively, in absolute ethanol I, II, III, and finally rinsed with distilled water. Antigen retrieval was performed using EDTA buffer under low heat for 10 min. The sections were allowed to cool naturally and washed with PBS on a shaker three times for 5 min each. To block endogenous peroxidase activity, the sections were incubated with 3% hydrogen peroxide at room temperature in the dark for 25 min, followed by another three PBS washes. For serum blocking, sections were incubated with 3% bovine serum albumin (BSA) (Servicebio, GC305010) at room temperature for 30 min. Subsequently, the primary antibody, anti-SERPING1 (Servicebio, GB112165; dilution 1:1000), was added dropwise, and sections were incubated overnight at 4 °C in a humidified chamber. After washing with PBS, the corresponding HRP-conjugated secondary antibody (S-vision Immunohistochemical Poly-Anti (*Goat Anti-Mouse*); Servicebio, G1301) was applied, and sections were incubated at room temperature for 50 min, followed by three PBS washes. Color development was achieved using freshly prepared DAB chromogenic solution (Servicebio, G1212), and the reaction time was monitored under a microscope. The reaction was terminated by rinsing with tap water. The nucleus was counterstained with hematoxylin for three minutes, then differentiated and counterstained, and then rinsed with running water. The sections were dehydrated and transparentized through a gradient ethanol series (75%, 85%, absolute ethanol I and absolute ethanol II), n-butanol, and xylene. After being slightly air-dried, the sections were mounted with neutral gum and observed under an optical microscope.

### 2.9 Mediation Analysis

#### 2.9.1 Upstream Mediation Analyses

We conducted a mediation analysis to investigate whether DNA methylation acts as a mediator through the expression of genes significantly associated with OP. The China National Center for Bioinformation serves as the national integrated platform for the centralized submission, secure storage, and open access of large-scale bioinformatics data in China, supporting interdisciplinary and translational biomedical research [51]. After identifying the obvious genes in MR and SMR, we obtain the potential probe IDs of DNA methylation arrays related to the obvious genes from the CNCB platform (https://ngdc.cncb.ac.cn/ewas/datahub/exploration). To explore the upstream regulatory mechanism, we obtained the mQTL data from the GoDMC (Genome-wide DNA Methylation Database from the Mendelian Randomization Perspective) platform (http://mqtldb.godmc.org.uk/index.php). GoDMC aggregates large-scale human genomic and epigenomic data generated from GWAS. These data are widely used to investigate the potential causal role of DNA methylation in complex traits and diseases. Extract the data of methylation sites for each positively correlated gene. Screen the single nucleotide polymorphism sites according to the *P* value less than 5e - 8, the window size of 10000kb, and the r^2^ less than 0.1.

**Fig. (1):**
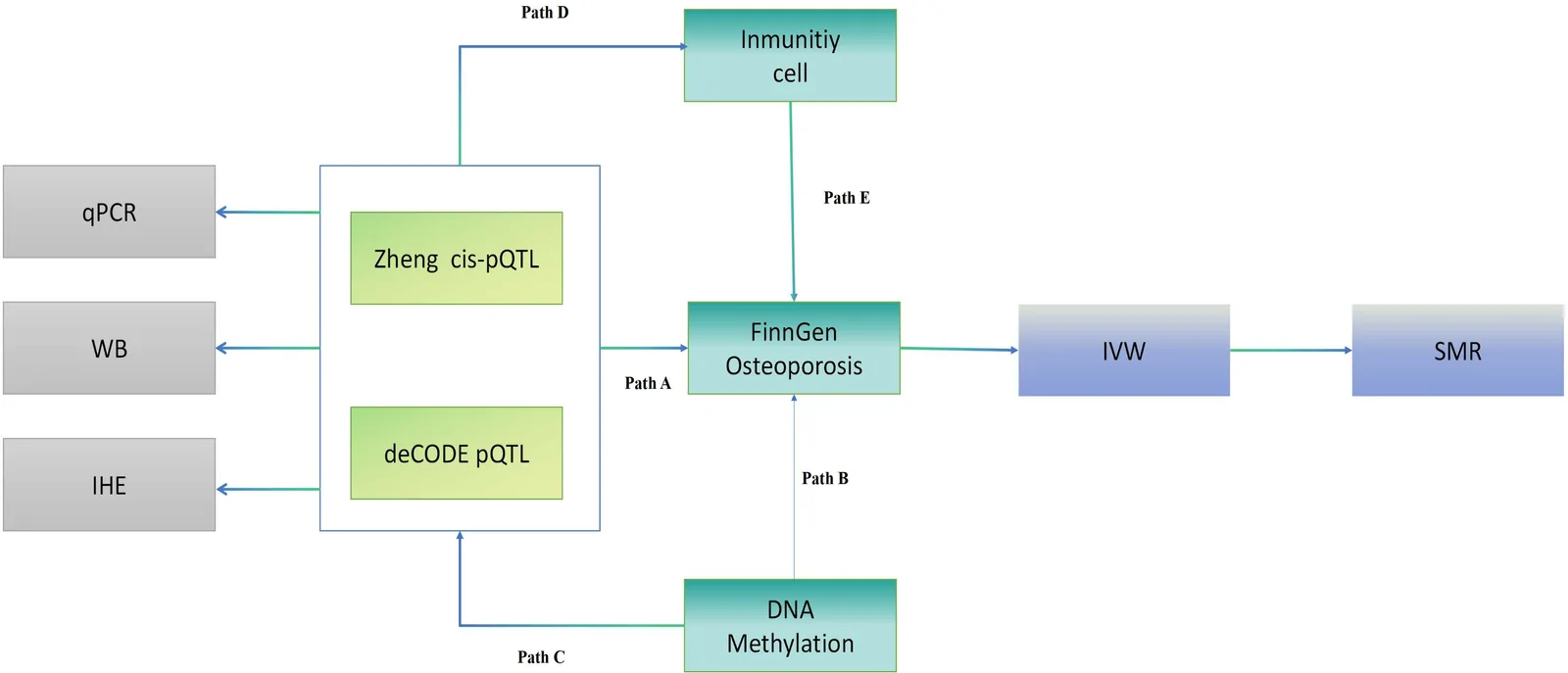
Technology Roadmap. Path (**A**) is the causal association between SERPINs and OP. Path (**B**) is the causal association between DNA methylation and OP. Path (**C**) is the association of DNA methylation with SERPINs. Path (**D**) is the association of SERPINs with immune cells. In Path E, there is a causal effect between immune cells and OP.

The mediation analysis was used to evaluate whether DNA methylation exerts an indirect regulatory effect on the relationship between protein expression and OP. First, the effect size of the genetic pQTL on OP was calculated and defined as Path A. Second, the effect size of the mQTL on OP was calculated and defined as Path B. Third, the effect size of the mQTL on the pQTL for genes showing significant associations was calculated and defined as Path C. The mediating effect (β_AC) was computed as the product of the coefficients for Path A and Path C, using the following formula: βAC=βA×βC. The mediation proportion was then calculated to quantify the percentage of the total effect mediated by DNA methylation, according to the equation: Mediation Proportion= (βAC/βB) × 100% (Fig. **1**).

#### 2.9.2 Downstream Mediation Analyses

Additional mediation analyses were conducted to examine whether genes with genetically supported associations with osteoporosis influence OP risk *via* changes in immune cell activity. Summary-level immune cell trait data were obtained from the IEU OpenGWAS Project (https://gwas.mrcieu.ac.uk/) [52], which provides harmonized GWAS results for a wide range of immunological phenotypes. This analysis aimed to evaluate whether alterations in gene expression mediated by DNA methylation contribute to OP development *via* changes in immune cell populations or functions. The framework for calculating effect size and the framework for calculating mediator effect follow the same method.

First, the effect size of the positive gene expression on immune cell traits was calculated and defined as Path D. Second, the effect size of immune cell traits on OP was calculated and defined as Path E. The mediating effect (β_DE) was determined as the product of the coefficients for Path D and Path E, using the formula: βDE=βD×βE.The mediation proportion—representing the proportion of the total genetic effect mediated by immune cell regulation—was calculated as: Mediation Proportion= (βDE/βA) × 100% (Fig. **1**).

## 3 RESULT

### 3.1 Technology Roadmap

As shown in Fig. (**1**), it presents the overall analysis process of this study.

### 3.2 MR Analysis of pQTL

Nine SERPIN family proteins were extracted from the cis-pQTL dataset reported by Zheng *et al* [44]*.* The “TwoSampleMR” package was used to evaluate the causal relationship between these proteins and OP. Since no eligible SNP met the requirements of two-sample Mendelian randomization (MR) screening, SERPINA11 was excluded. The result was that four SERPIN proteins - SERPINA1, SERPINA4, SERPINA10, SERPING1 - had obvious causal associations with OP(*P *< 0.05) Table (**1**).

**Fig. (2):**
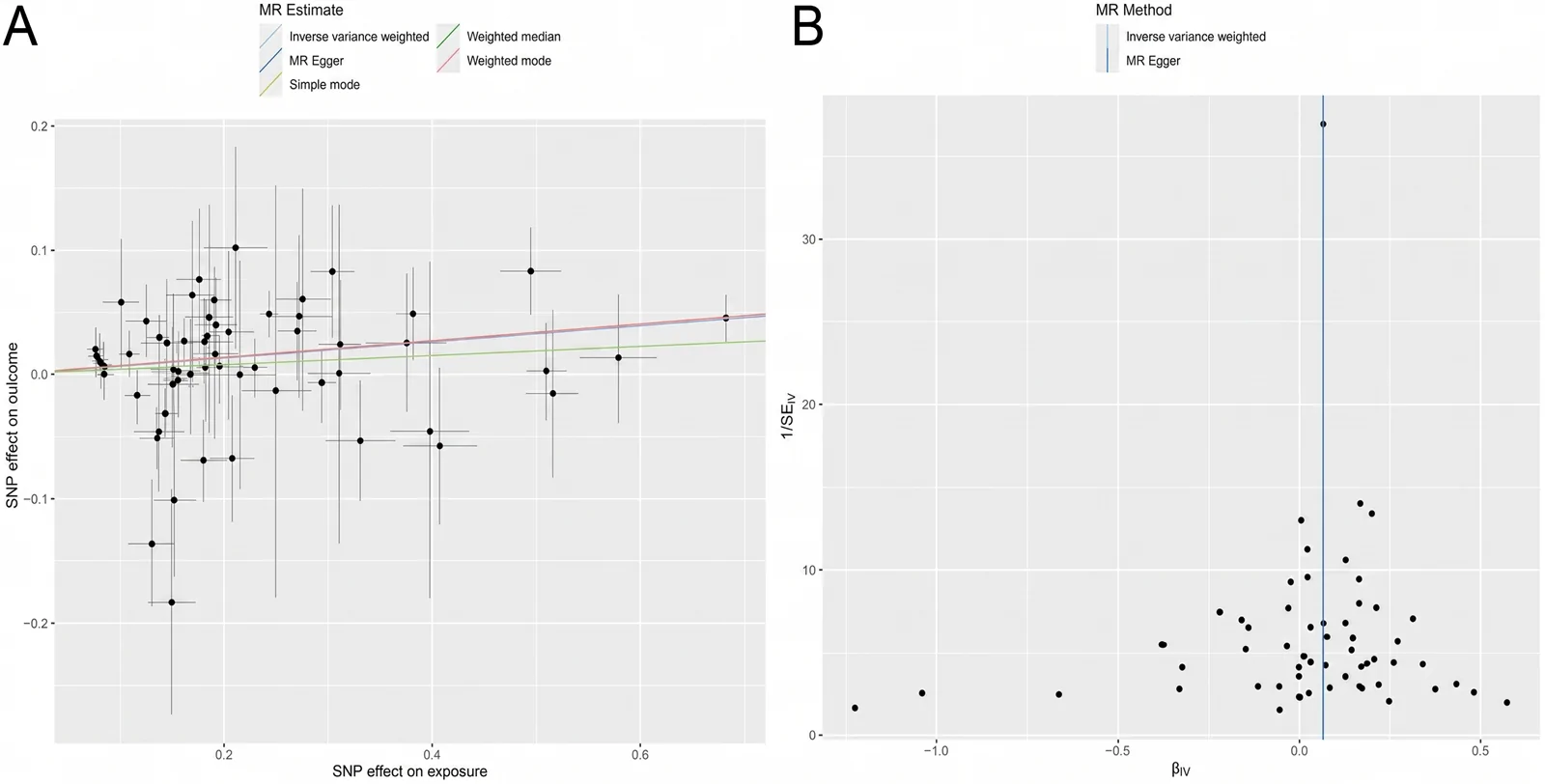
Scatter plot of causal effects and funnel plot of heterogeneity test for the association between SERPING1 and OP. Figure Legend: (**A**) : This figure shows the relationship between the true SNP effect value and the estimated value between models. The X-axis is the true SNP effect value, and the Y-axis is the corresponding estimated effect value. Different line and point styles represent different models, and the estimation accuracy and performance when recovering the true SNP effect can be compared. (**B**) . This panel compares the performance of different expectation–maximization (EM) methods based on bias (x-axis) and mean square error (y-axis). Each point represents an EM method. The joint distribution of bias and mean square error shows the performance differences of different methods. The offset reference is the vertical blue line, which is used to compare the accuracy of the estimate of the EM approximation.

Initial MR analysis found that there was one or two SNPs in 4 SERPIN proteins, and sensitivity analysis could not be carried out. Then, the pQTL data of these 4 proteins were obtained from the deCODE database, and MR analysis was re-conducted for verification using the same screening criteria as the cis-pQTL data set. The F-values of all SNPs in the calculations all exceed 10, which indicates that the selected instrumental variables were relatively strong and the bias of weak instrumental variables was unlikely to occur. Bonferroni correction was used for multiple tests. The adjusted *P*-values (*P.Adjust < 0.05*) showed that there was a causal association between exposure and outcome. For certain SERPIN family members, no cis-pQTLs meeting the predefined instrument selection criteria were available in the deCODE dataset. Therefore, these proteins were not included in subsequent MR analyses. Following validation, only SERPING1 exhibited a significant causal relationship with OP (*P. Adjust*< 0.05) (Table **2** and **3**; Fig. **2**).

### 3.3 Sensitivity Analysis

**Fig. (3):**
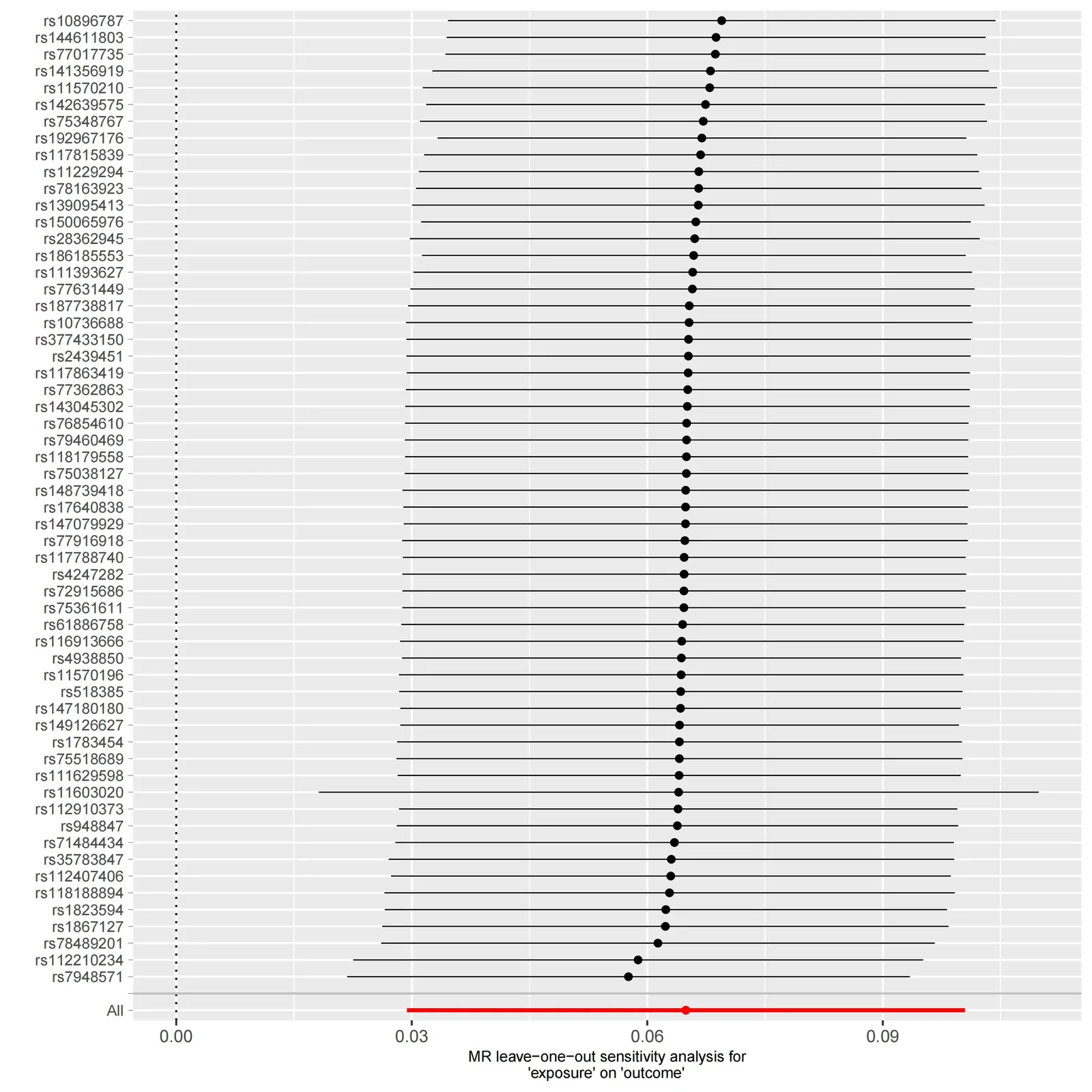
Leave-one-out sensitivity analysis for SERPING1-OP association in MR. Leave-one-out sensitivity analysis of osteoporosis-related SNPs. The horizontal axis is the confidence interval of the causal estimate when each SNP is excluded; the vertical axis lists the identifiers of each SNP. Each black dot is the causal estimate after excluding one single nucleotide polymorphism; the red vertical line is the estimate of the overall causal effect. The point distribution relative to the reference line is used to evaluate whether a single SNP has an improper impact on the association between genetic tools and osteoporosis.

To verify the stability of the MR results, the TwoSampleMR package was used to check through heterogeneity analysis, horizontal pleiotropy assessment, and leave-one-out sensitivity analysis. The results showed that the research results were not affected by heterogeneity or horizontal pleiotropy, and the selected instrumental variables were effective. In addition, in the leave-one-out analysis, removing any one SNP had little impact on the overall causal estimate, confirming the stability and reliability of the final results (Table **4**; Fig. **3**).

### 3.4 SMR Analysis

A SMR analysis was performed to support the causal relationship in the MR results. The HEIDI test was used to check whether horizontal pleiotropy had an impact on the association. The result showed that P_SMR was less than 0.05, which indicated that there was a significant causal association between SERPING1 expression and OP. And P_HEIDI was greater than 0.05. This association is not affected by horizontal pleiotropy, and the result is stable and reliable (Table **5**).

### 3.5 qPCR/Western Blot/IHC

**Fig. (4):**
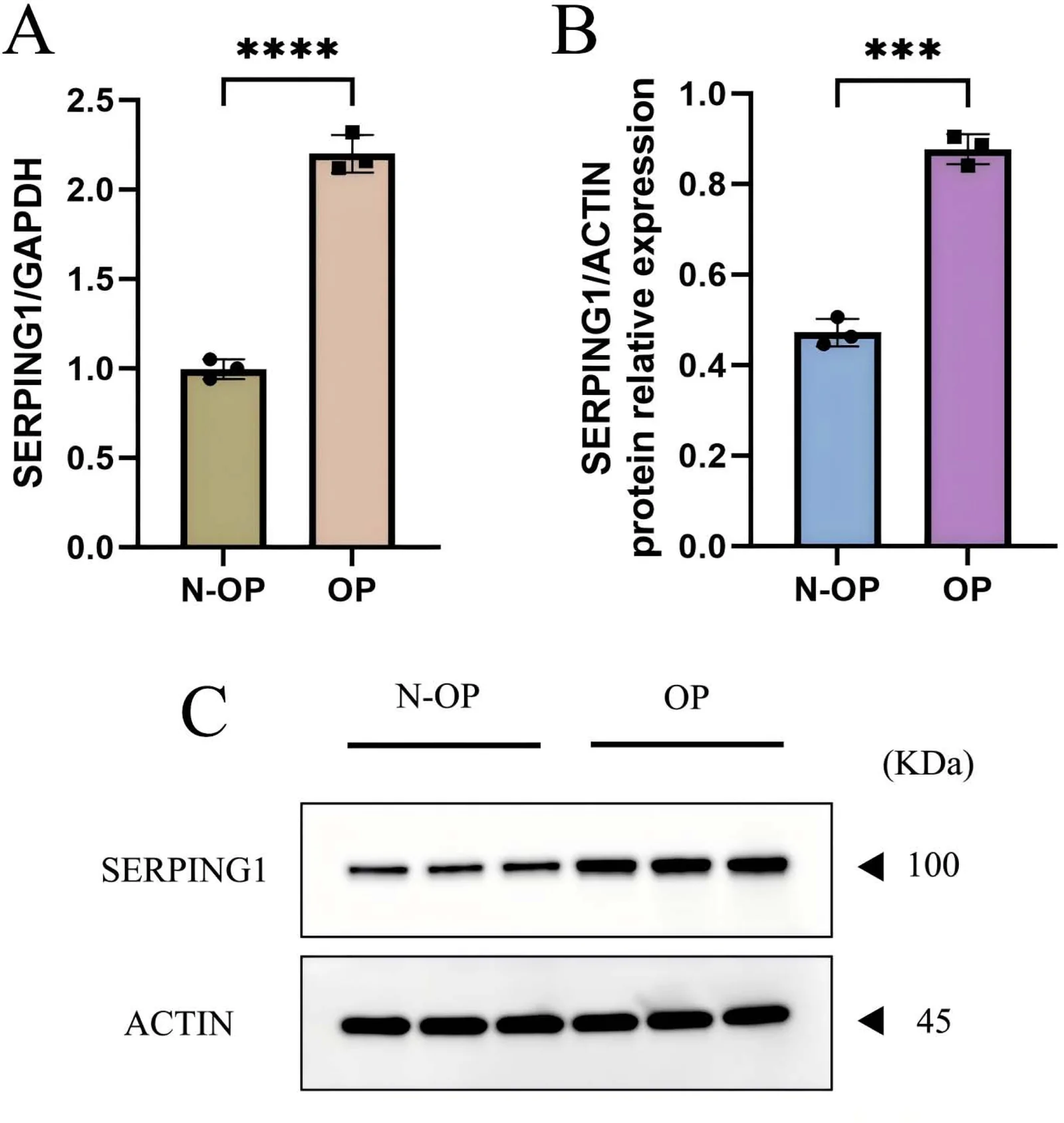
Validation of SERPING1 expression in bone tissue of OP and N-OP patients. (**A**) mRNA expression levels of SERPING1 determined by qPCR. Relative expression levels of the SERPING1 gene in the non-osteoporosis (N-OP) and osteoporosis (OP) groups, with GAPDH used as the internal control. The horizontal axis denotes the N-OP and OP groups, and the vertical axis represents the relative SERPING1/GAPDH expression level. (**B**) Relative protein expression levels of SERPING1 quantified by Western blot analysis. Relative protein expression levels of SERPING1 in the N-OP and OP groups, with ACTIN used as the internal control. The horizontal axis indicates the N-OP and OP groups, while the vertical axis represents the relative SERPING1/ACTIN protein expression level. (**C**) Representative Western blot images showing SERPING1 protein expression in OP and N-OP groups (****P < 0.0001; ***P < 0.001).

**Fig. (5):**
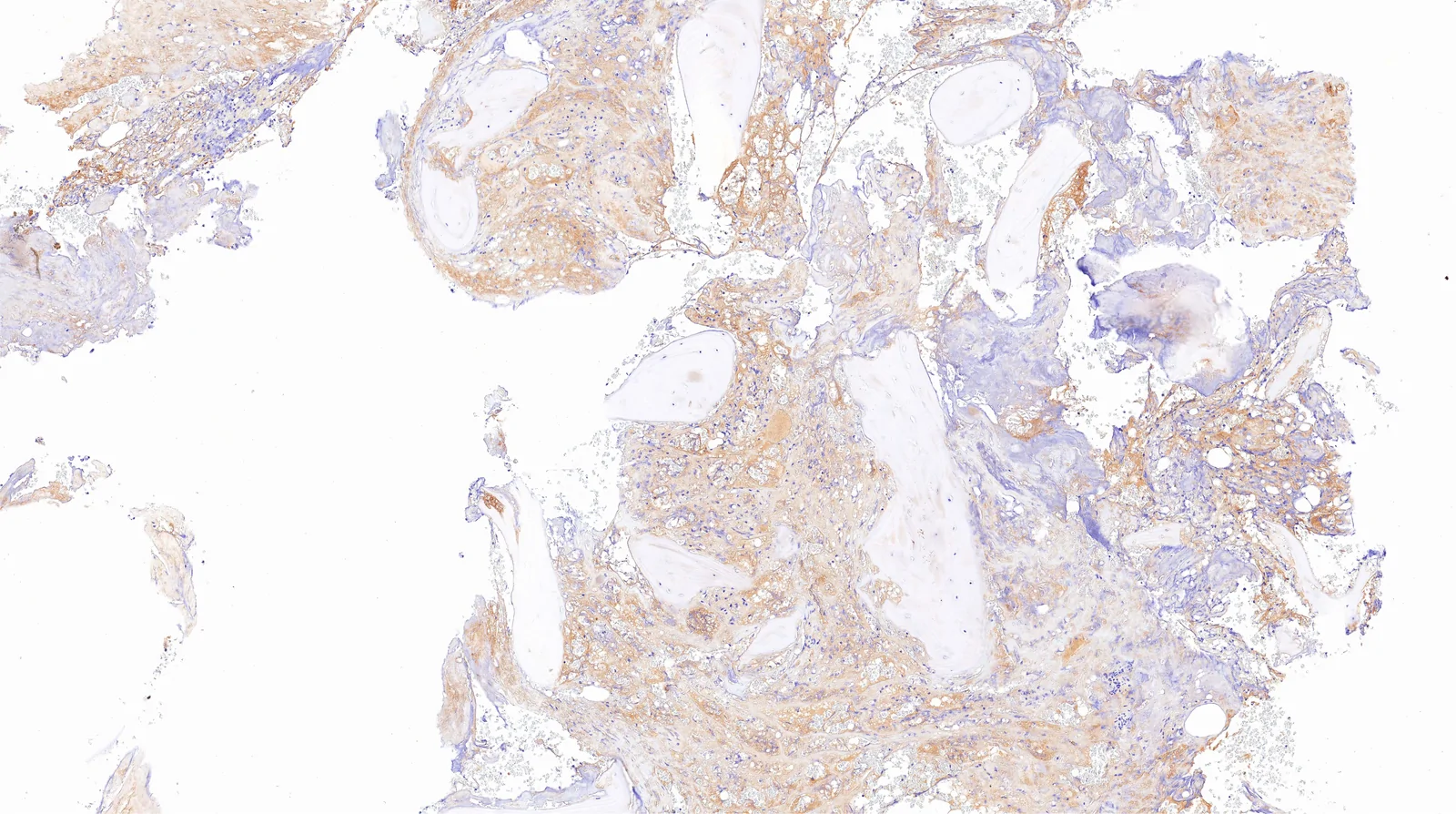
IHC analysis of SERPING1 expression in bone tissue from osteoporosis (OP) patients (magnification, 5×). A large number of fragmented bone trabeculae are visible within the tissue, accompanied by extensive brownish-yellow positive staining surrounding the fragmented regions. The positive immunoreactivity for SERPING1 is indicated by the brownish-yellow coloration, demonstrating strong SERPING1 protein expression in OP bone tissue.

**Fig. (6):**
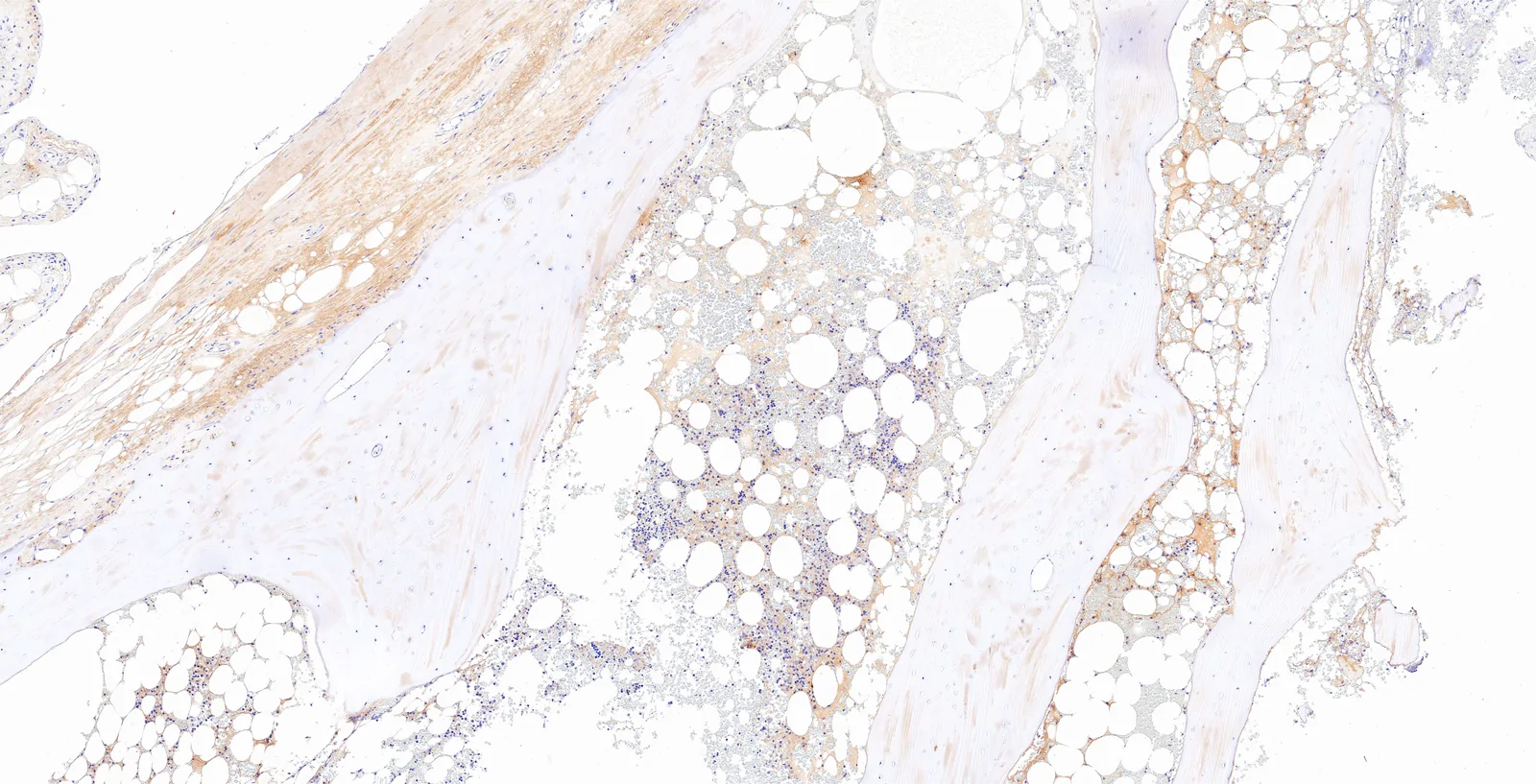
IHC analysis of SERPING1 expression in bone tissue from N-OP patients (magnification, 5×). The bone tissue exhibits intact and dense trabecular structures with a well-preserved medullary cavity containing partial adipose tissue. Only minimal background staining is observed, and brownish-yellow positive signals, indicative of SERPING1 expression, are nearly absent. These findings demonstrate low SERPING1 protein expression in N-OP bone tissue compared with OP samples.

Samples of patients with OP and (N-OP controls were collected to study the role of SERPING1 in bone tissue. qPCR and WB tests were performed at the mRNA and protein levels. The results showed that the expression of SERPING1 mRNA in the OP group was higher than that in the N-OP control group (*P *< 0.05) (Figs. **4A**-**C**). Tissue pathological analysis of samples from OP patients showed typical osteoporosis degeneration, with an increase in bone fragments and damage to the microstructure of trabecular bone. IHC staining showed strong brownish-yellow diffuse positive signals for SERPING1 protein surrounding the fragmented bone regions in OP tissue, whereas N-OP bone samples exhibited intact bone structures and medullary cavities with only minimal background staining (Figs. **5** and **6**). Integrated qPCR, Western blot, and IHC analyses consistently confirmed that SERPING1 expression was markedly elevated in OP bone tissue, thereby validating the MR findings and supporting a potential role of SERPING1 in OP pathogenesis.

### 3.6 Upstream Mediation Analyses

The DNA methylation site information for SERPING1 was obtained from the CNCB database, and the corresponding mQTLs were downloaded from the GoDMC platform. A two-sample MR analysis was performed using the IVW method to assess potential causal relationships between DNA methylation sites, SERPING1 expression, and OP. The results showed that only one methylation site, cg15918732, exhibited a statistically significant effect on both SERPING1 expression and OP.

Specifically, the effect size (β B) for the association between cg15918732 methylation and OP was 0.12 (*P* = 0.0009 < 0.05), and the effect size (β C) for the association between cg15918732 methylation and SERPING1 expression was 1.67 (*P*=4.32e-228 < 0.05). Mediation analysis suggested that cg15918732 DNA methylation may be associated with SERPING1 expression and could partially account for the genetically inferred association with OP, with an estimated mediation proportion of approximately 88.9% (Table **6**).

### 3.7 Downstream Mediation Analyses

Immune cell data were obtained from the IEU Open GWAS Project, and a two-sample MR analysis was conducted using the IVW method to assess the causal relationships between SERPING1, immune cell types, and OP. Based on the MR results, three immune cell types—TBNK (CD16^-^ CD56^+^ on NKs), Monocytes (HLA DR^+^ on CD14^-^ CD16^-/+^), and maturation stages of T cells (CD4 on CD4^+^)—showed significant causal associations with OP. The P-values from MR analyses between SERPING1 and these immune cells were all < 0.05, confirming statistical significance. The estimated effect sizes (βD) between SERPING1 and each immune cell type were as follows: TBNK, β = −0.07 (*P*=0.004); Monocyte, β = 0.05 (*P* = 0.004); and Maturation stage of T cell, β = −0.08 (*P* = 0.009). Mediation analyses suggested that these three immune cell traits may be involved in the genetically inferred association between SERPING1 expression and OP, with estimated mediation proportions of 4.4%, 3.3%, and 13.2% (Table **7**).

## 4 DISCUSSION

In this study, two-sample MR, SMR, and mediation analyses were employed to investigate genetically inferred associations between SERPIN family genes and OP. Among these genes, only the expression of SERPING1 showed a consistent positive correlation with OP risk in both MR and SMR analyses. Further analysis revealed that the expression of SERPING1 might be related to changes in DNA methylation and immune-related traits, suggesting potential upstream and downstream regulatory relationships. To support the research results based on MR and SMR, the clinical bone tissue samples were analyzed, and the expression of SERPING1 was evaluated by qPCR, Western blot, and IHC. All the detection results were the same, and the expression of SERPING1 in the bone samples of OP patients was up-regulated. This study systematically explored the inferred association between genes of SERPING1 and OP, suggesting that the epigenetic regulation of SERPING1 might be related to OP due to changes in the immune cell profiles. These results provided important clues for understanding the molecular mechanisms underlying OP and might inform future research into candidate biomarkers and therapeutic strategies.

Initially, four members of the SERPIN family (SERPINA1, SERPINA4, SERPINA10, SERPING1) were found by MR screening, and they were speculated to be related to OP. The second verification used the deCODE database. After Bonferroni correction, only SERPING1 was statistically significant, and the corrected P value was 0.0012. Furthermore, SMR analysis supported the robustness of the SERPING1 association, with no evidence of horizontal pleiotropy (P_HEIDI = 0.149). SERPINA1 and SERPINA10 were excluded from further analyses because no sufficient valid cis-pQTL instruments meeting genome-wide significance and linkage disequilibrium criteria were available in the deCODE dataset. Although SERPINA4 exhibited a nominal association with OP, the association did not reach statistical significance (*P* = 0.098 > 0.05). Therefore, subsequent analyses focused on SERPING1 as the most robust and reliably instrumented SERPIN associated with OP.

Our MR and SMR analyses indicated that genetically predicted SERPING1 expression is positively associated with OP risk. SERPING1 belongs to the SERPIN superfamily and encodes the C1 esterase inhibitor (C1-INH), a plasma protein involved in complement, coagulation, and inflammatory regulation [53]. C1-INH is a circulating glycoprotein with well-characterized structural and biochemical properties [54]. Loss-of-function mutations in SERPING1 cause C1-INH deficiency and are responsible for hereditary angioedema (HAE) [55, 56]. Beyond HAE, genetic variation in SERPING1 has been associated with several immune-related and inflammatory diseases [57-59]. However, the role of SERPING1 in OP remains poorly understood, with limited evidence available in bone-related contexts. Research on non-bone diseases may provide mechanistic hypotheses for the function of SERPING1. In lung cancer models, SERPING1 overexpression is associated with abnormal mTOR-related signaling pathways. These findings suggest that SERPING1 may be linked to autophagy-related pathways in certain disease settings [60]. Autophagy dysfunction, often accompanied by oxidative stress, has been implicated in impaired osteoblast function and reduced bone mineral density in osteoporosis [61, 62]. Evidence from non-bone tissues suggests that increased SERPING1 expression may be associated with alterations in mTOR signaling and autophagic activity, providing a biologically plausible link to these processes [63-65]. These studies provide a biological background, but not direct evidence for mechanisms.

Downstream mediation analysis showed that genetically predicted SERPING1 expression might be related to changes in specific immune-related traits, with reduced TBNK, increased abundance of mature-stage T cells, and increased monocyte levels, among which the mediation proportions of 4.4%, 13.2%, and 3.3%. These immune cell populations have been implicated in osteoimmune interactions and bone remodeling in previous studies, supporting the biological plausibility of an immune-related component in the SERPING1–OP association [66-69]. Notably, SERPING1 expression has been reported in immune cells, including monocytes, in inflammatory disease contexts, providing a potential link between SERPING1 expression and alterations in immune cell profiles [70, 71]. However, immune cell function and cytokine activity were not directly assessed in bone tissue in the present study, and the immune-related associations identified here are derived from MR-based mediation analyses rather than direct experimental validation. Therefore, these findings are hypothetical, and further experimental studies are required to clarify whether and how SERPING1 may influence immune–bone crosstalk in osteoporosis. Mediation proportions should be interpreted with caution, given differences in tissue context and measurement scales across data sources.

Upstream mediation analysis suggested that cg15918732 DNA methylation may be associated with SERPING1 expression and genetically inferred osteoporosis risk. The large effect size observed for cg15918732 methylation in relation to SERPING1 expression may be attributable to strong cis-mQTL effects in blood-based datasets and may not proportionally reflect biological effects in bone tissue. Previous studies have reported that alterations in DNA methylation of bone-related genes are linked to osteoporosis, supporting the biological plausibility of epigenetic regulation in bone metabolism [64, 72]. Moreover, the demethylation of CpG in immune regulatory genes that are related to diseases is directly detected in human tissues, such as increased FOXP3 demethylation observed in colorectal cancer [73] and cholangiocarcinoma samples [74], which further offers support for the biological rationality of epigenetic regulation in immune-mediated diseases.

To our knowledge, the relationship between cg15918732 methylation and osteoporosis has yet to be reported. The present study provides initial genetically inferred evidence suggesting a potential link based on an mQTL-driven MR study and mediation analyses. The cg15918732 site is located in close genomic proximity to the SERPING1 gene on chromosome 11 [75], supporting the plausibility of a regulatory relationship between this CpG locus and SERPING1 expression. However, DNA methylation at the cg15918732 site was not directly measured in bone tissue. The inferred regulatory relationship is therefore based on genetic inference rather than experimental validation. Whether the regulatory role of cg15918732 is definite, for example, whether it is in the promoter, enhancer, or other regulatory elements, cannot be determined from the current data. Therefore, the methylation status of cg15918732, as well as the expression status of SERPING1, and the association formed with osteoporosis should be regarded as hypothesis generation. Future studies incorporating direct methylation profiling and functional epigenetic assays will be required to elucidate the mechanistic role of cg15918732 in osteoporosis.

## 5 STRENGTHS OF THIS STUDY

To our knowledge, this study provides the first genetically inferred evidence suggesting a potential association among DNA methylation, SERPING1 expression, and OP. By applying an MR study, we aimed to reduce confounding and reverse causality, which are common limitations of traditional observational studies. Furthermore, by integrating SMR with qPCR, Western blotting, and immunohistochemistry, we provided supportive evidence for the genetically inferred association between SERPING1 expression and OP. Through mediation analyses, we explored potential upstream and downstream pathways linking SERPING1 to OP, including DNA methylation and alterations in immune cell profiles, generating hypotheses for future mechanistic investigation. This integrative analytical framework strengthened the consistency of the findings across multiple data sources and analytical approaches. In addition, the large-scale dataset used in this study—including 7,300 OP cases and 358,014 controls from the FinnGen database—provided adequate statistical power to detect modest genetic associations. The inclusion of genetic and proteomic data from multiple independent databases increased the consistency of the observed associations across datasets.

## 6 SHORTCOMINGS AND LIMITATIONS

There are several limitations in this study. Firstly, the outcome data were from the Finnish population, which may limit the generalizability of the findings to other racial and genetic backgrounds. Secondly, this study lacked cell and animal experiments to directly confirm the effect of methylation of cg15918732 on SERPING1 expression and downstream immune pathways. Third, the ex vivo bone tissue cohort was small, primarily due to limited specimen availability. It should be noted that this design was for exploratory purposes, not for statistical hypothesis testing. These samples were included to provide qualitative support for the direction of SERPING1 expression inferred from MR and SMR analyses and should be interpreted with caution. Accordingly, future studies in larger and more diverse populations, together with functional investigations, will be required to further evaluate these genetically inferred associations and their biological relevance.

## CONCLUSION

This study shows that DNA methylation of cg15918732 may be associated with SERPING1 expression in osteoporosis and immune-related traits. These findings provide insights into potential epigenetic and immunological pathways in osteoporosis and may also contribute to future mechanistic and translational research. However, further experimental studies are required to validate these genetically inferred associations and to clarify the biological functions and regulatory mechanisms of SERPING1 in bone metabolism and OP.

## Figures and Tables

**Table 1:** Estimated causal effects of SERPIN family proteins on OP based on MR analysis using cis-pQTL data from *Zheng *et al*.’s* study.

Exposure	Outcome	b	se	OR (95% CI)	*P*	Method
**SERPINA1**	**OSTEOPOROSIS**	**-0.10**	**0.04**	**0.91**	**0.03**	**Wald Ratio**
SERPINA3	OSTEOPOROSIS	-0.07	0.06	0.92	0.20	Wald ratio
**SERPINA4**	**OSTEOPOROSIS**	**-0.07**	**0.03**	**0.94**	**0.02**	**IVW**
**SERPINA10**	**OSTEOPOROSIS**	**0.04**	**0.02**	**1.05**	**0.03**	**Wald ratio**
SERPINA11	OSTEOPOROSIS	NA	NA	NA	NA	NA
SERPINF1	OSTEOPOROSIS	-0.05	0.04	0.95	0.16	Wald ratio
SERPINF2	OSTEOPOROSIS	0.0007	0.09	1.00	0.99	Wald ratio
**SERPING1**	**OSTEOPOROSIS**	**0.08**	**0.03**	**1.08**	**0.01**	**Wald ratio**
SERPINE2	OSTEOPOROSIS	0.026	0.05	1.02	0.60	Wald ratio

**Table 2:** Estimated causal effects of SERPIN family proteins on OP based on MR analysis using pQTL data from the deCODE database.

Exposure	Outcome	nsnp	b	se	OR (95% CI)	*P*	*P.Adjust*	Method
**SERPING1**	**OSTEOPOROSIS**	**58**	**0.06**	**0.02**	**1.06**	**0.0003**	**0.0012**	**IVW**
SERPINA1	OSTEOPOROSIS	NA	NA	NA	NA	NA	NA	NA
SERPINA4	OSTEOPOROSIS	95	-0.05	0.03	0.95	0.098	0.392	IVW
SERPINA10	OSTEOPOROSIS	NA	NA	NA	NA	NA	NA	NA

**Table 3:** Estimated causal effects of SERPING1 on OP pathogenesis based on MR analysis using pQTL data from the deCODE database.

Exposure	Outcome	Method	nsnp	b	se	*P*	OR (95% CI)
SERPING1	OSTEOPOROSIS	MR Egger	58.00	0.07	0.03	0.04	1.07
		Weighted median	58.00	0.07	0.03	0.01	1.07
		IVW	58.00	0.06	0.02	0.0003	1.06
		Simple mode	58.00	0.04	0.05	0.49	1.04
		Weighted mode	58.00	0.07	0.02	0.01	1.07

**Table 4:** Results of heterogeneity and pleiotropy tests in MR analysis for the association between SERPING1 and OP.

Exposure	Outcome	Q	Q_df	Q_pval	egger_intercept	se	pval
SERPING1	OSTEOPOROSIS	65.92	57	0.20	-0.0003	0.007	0.97

**Table 5:** Results of SMR analysis for the association between SERPING1 and OP.

Gene	Exposure	Outcome	topSNP	nsnp_HEIDI	*P_SMR*	*P_HEIDI*
ENSG00000149131	SERPING1	OSTEOPOROSIS	rs28362951	20	8.000709e-03	1.493794e-01

**Table 6:** Mediation analysis results of DNA methylation sites, SERPING1, and OP.

CpG Sites	β C	Path C pval	β A	β AB	β B	Path B pval	Estimated Mediation Proportion
cg15918732	1.67	4.32e-228	0.06	0.11	0.12	0.0009	88.9%

**Table 7:** Mediation analysis results of immune cell types, SERPING1 gene expression, and OP risk.

id	Immune Cells	β D	Path D pval	β A	Total Effect β DE	β E	Path E pval	Estimated Mediation Proportion
ebi-a-GCST90001884	TBNK (CD16-CD56 on NK)	-0.07	0.004	0.06	0.003	-0.04	0.01	4.4%
ebi-a-GCST90002009	Monocyte (HLA DR on CD14- CD16-+)	0.05	0.019	0.06	0.002	0.04	0.02	3.3%
ebi-a-GCST90002022	Maturation stages of T cell (CD4 on CD4+)	-0.08	0.009	0.06	0.009	-0.10	0.03	13.2%

## Data Availability

The data that support the findings of this study are available from the corresponding author upon reasonable request.

## LIST OF ABBREVIATIONS

AT: Antithrombin
BCA: Bicinchoninic Acid
BMD: Bone Mineral Density
BMP2: Bone Morphogenetic Protein 2
C1-INH: C1 Esterase Inhibitor
CNCB: China National Center for Bioinformation
CpG: Cytosine–phosphate–guanine
DNA: Deoxyribonucleic Acid
GWAS: Genome-wide Association Study
HAE: Hereditary Angioedema
IHC: Immunohistochemistry
IL-17A: Interleukin-17A
IL-4: Interleukin-4
IVW: Inverse-variance Weighted
LD: Linkage Disequilibrium
mQTL: Methylation Quantitative Trait Locus
MR: Mendelian Randomization
MSC: Mesenchymal Stem Cell
NK cell: Natural Killer Cell
NKT cell: Natural Killer T Cell
OP: Osteoporosis
OPG: Osteoprotegerin
PAI-1: Plasminogen Activator Inhibitor-1
PCOS: Polycystic Ovary Syndrome
pQTL: Protein Quantitative Trait Locus
qPCR: Quantitative real-time Polymerase Chain Reaction
RANKL: Receptor Activator of Nuclear Factor-κB Ligand
RCL: Reactive Center Loop
ROS: Reactive Oxygen Species
RUNX2: Runt-related Transcription factor 2
SERPIN: Serine Protease Inhibitor
SMR: Summary-data-based Mendelian Randomization
SNP: Single Nucleotide Polymorphism
SP7: Osterix
TBNK cell: T Cell, B Cell, And Natural Killer Cell
TNF-α: Tumor Necrosis Factor-α
WB: Western Blotting
5mC: 5-methylcytosine

## STANDARDS OF REPORTING

STROBE-MR guidelines were followed.
